# Normal tissue complication probability model of temporal lobe injury following re-irradiation of IMRT for local recurrent nasopharyngeal carcinoma

**DOI:** 10.3389/fonc.2024.1394111

**Published:** 2024-05-30

**Authors:** Xiyin Guan, Jiyou Peng, Jiayao Sun, Xing Xing, Chaosu Hu

**Affiliations:** ^1^ Department of Radiation Oncology, Shanghai Proton and Heavy Ion Center, Fudan University Cancer Hospital, Shanghai, China; ^2^ Shanghai Key Laboratory of Radiation Oncology, Shanghai, China; ^3^ Shanghai Engineering Research Center of Proton and Heavy Ion Radiation Therapy, Shanghai, China; ^4^ Department of Radiation Oncology, Fudan University Shanghai Cancer Center, Shanghai, China; ^5^ Department of Oncology, Shanghai Medical College, Fudan University, Shanghai, China

**Keywords:** recurrent nasopharyngeal carcinoma, intensity modulated radiotherapy, re-irradiation, temporal lobe injury, normal tissue complication probability model

## Abstract

**Purpose:**

We tried to establish the normal tissue complication probability (NTCP) model of temporal lobe injury of recurrent nasopharyngeal carcinoma (NPC) patients after two courses of intensity modulated radiotherapy (IMRT) to provide more reliable dose-volume data reference to set the temporal lobe tolerance dose for recurrent NPC patients in the future.

**Methods and materials:**

Recurrent NPC patients were randomly divided into training data set and validation data set in a ratio of 2:1, All the temporal lobes (TLs) were re-contoured as R/L structures and named separately in the MIM system. The dose distribution of the initial IMRT plan was deformed into the second course planning CT via MIM software to get the deformed dose. Equivalent dose of TLs in 2Gy fractions was calculated via linear quadratic model, using an α/β=3 for temporal lobes. NTCP model that correlated the irradiated volume of the temporal lobe and? the clinical variables were evaluated in a multivariate prediction model using AUC analysis.

**Results:**

From Jan. 2010 to Dec. 2020, 78 patients were enrolled into our study. Among which 26 (33.3%) developed TLI. The most important factors affecting TLI was the sum-dose d1.5cc of TL, while the possible clinical factors did not reach statistically significant differences in multivariate analysis. According to NTCP model, the TD5 and TD50 EQD2 dose of sum-dose d1.5cc were 65.26Gy (46.72–80.69Gy) and 125.25Gy (89.51–152.18Gy), respectively. For the accumulated EQD2 dose, the area under ROC shadow was 0.8702 (0.7577–0.9828) in model validation, p<0.001.

**Conclusion:**

In this study, a NTCP model of temporal lobe injury after a second course of IMRT for recurrent nasopharyngeal carcinoma was established. TD5 and TD50 doses of temporal lobe injury after re-RT were obtained according to the model, and the model was verified by validation set data.

## Introduction

Nasopharyngeal carcinoma (NPC) is prevalent among Asians, particularly in Southern China, and is epidemiologically linked to Epstein-Barr virus infection, where the age-standardized incidence ranges from 15 to 50 cases per 100,000 population (1, 2). Now Intensity modulated radiotherapy (IMRT) is widely used as the primary treatment modality for non-metastasis NPC due to its anatomic location and radio-sensitivity. Local recurrence remains one of the common patterns of treatment failure. Overall, 10% to 20% local failures occur after definitive IMRT. Surgery is a preferred choice for small resectable superficial recurrent lesion (3, 4). Re-irradiation with IMRT remains the mainstay of treatment for advanced stage recurrence. However, due to the considerable critical organs surrounding the tumor, re-irradiation may lead to severe toxic side effects.

Temporal lobe injury (TLI) is a common late complication after re-irradiation for recurrent nasopharyngeal carcinoma, which is often manifested as memory decline, cognitive dysfunction, motor dysfunction, emotional disorders, language disorders, and other related symptoms, leading to a decline in the quality of life. IMRT can effectively limit the high-dose exposure of the temporal lobe. The TLI probability after the first course radiotherapy was about 4.6–16% (5–7). However, in recurrent nasopharyngeal carcinoma, especially for patients with large tumor volume, especially those with skull base invasion or intracranial invasion, a second course of high dose irradiation would be necessary, thus TLI is inevitable (8–10). Currently, there is very limited experience in determining the dose-volume tolerance of the temporal lobe for a second course of radiotherapy. In this study, we retrieved the first and second course of IMRT plan data for recurrent nasopharyngeal carcinoma patients and established the NTCP model of TLI based on clinical and dosimetric parameters. We believe this study would provide a more reliable reference for dose-volume data, and would provide assistance in the decision of temporal lobe dose limitation in the future.

## Method and materials

### Inclusion and exclusion criteria

Inclusion criteria included:(1) Recurrent nasopharyngeal carcinoma confirmed by pathology or at least two imaging methods; (2) Both of the two courses of radiotherapy were using IMRT techniques, and the 2 courses of radiation plans were attainable; (3) Distant metastasis was excluded by chest CT, abdominal ultrasound, emission CT bone scan or whole body positron emission tomography-computed tomography (PET-CT); (4) Karnofsky performance scale (KPS) score ≥70; (5) Patients received complete 2 courses radiotherapy; (6) Patients were regularly followed up in the outpatient clinic with complete magnetic resonance images for at least every 6 months. Exclusion criteria included: (1) TLI occurred before the second course of radiotherapy; (2) The follow up time was less than 6 months; (3) Patient was unable to receive MRI to accurately assess TLI; (4) TLI cannot be differentiated from tumor progression or recurrence; (5) The two courses radiotherapy plan cannot be obtained completely.

### Immobilization and treatment plan

All initial and re-irradiation plans were obtained in Fudan University Shanghai Cancer Center. All patients can proceed to the immobilization, planning, and treatment process only after signing the informed consent for radiotherapy. Patient was immobilized in the supine position with a thermoplastic mask. CT was performed with slice thickness of 5mm after immobilization, ranging from1.5cm above the cranial vertex to at least 2cm below the sternoclavicular joint. The target volumes were delineated on CT images using Pinnacle (Pinnacle 3; Philips Corp, Fitchburg, WI) treatment planning system. Inverse IMRT plans were optimized using Pinnacle. For the initial course IMRT, the total dose to primary tumor was 66 Gy in 30 fractions for T1 or T2 stage disease, and 70.4 Gy in 32 fractions for T3 or T4. A total dose of 60 Gy and 54 Gy was delivered to the high-risk and low-risk clinical tumor volume (CTV) in 30–32 fractions, respectively. For the re-irradiation course, only recurrent tumor and the positive involved lymph node regions were irradiated. The prescribed doses were 60–70 Gy to the gross tumor volume (GTV)and 50–60 Gy to the CTV, delivered in 25–35 fractions. The normal tissue constraints and plan evaluation were in accordance with the Radiation Therapy Oncology Group 0225 protocol. All the radiation were delivered using a simultaneous integrated boost-IMRT technique using Pinnacle. Patients with advanced T stage disease or positive lymph nodes received cisplatin-based induction or concurrent chemotherapy during IMRT.

### Image assessment and diagnostic criteria for TLIs

All the TLIs were diagnosed based on MRI findings. These abnormalities were verified by two radiologists, and any dis-agreements were resolved by consensus. Residual or progressive disease was excluded when determining the TLI site. The diagnostic criteria for TLI were as follows: (1) contrast-enhanced lesions, lesions with spotted or patchy enhancement with or without necrosis on post-contrast T1-weighted images; (2) white matter lesions, increased signal intensity on T2-weighted images in white matter; (3) cysts, round or oval lesions of very high signal intensity on T2-weighted images with a thin or imperceptible wall.

### Dose volume histogram data calculation

Both the two courses IMRT plan were imported into the MIM system (MIM software v6.5.9, Cleveland, OH, USA). To ensure precise delineation of the temporal lobe, all temporal lobes were re-contoured by the physician in the re-irradiation plan as R/L structures and named separately using MIM software and cross-checked by another experienced physician. In cases where the tumor infiltrated into the intracranial tissue, this specific region of the temporal lobe was delineated as normal tissue. The dose distribution of the first IMRT plan was deformed into the planning CT of the re-radiotherapy via MIM to get the deformed dose. Since the fractionation of the two IMRT plans were not identical, equivalent dose in 2Gy fractions was calculated via linear quadratic model, using an α/β=3 for temporal lobes. The equivalent dose ( $EQD_{3}^{2}$ ) of the deformed dose and the dose of re-radiotherapy was accumulated to obtain the accumulated dose based on former registration via Python program (v3.9.6). The dose volume histograms of the bilateral temporal lobes and the TLI of the deformed dose, re-irradiated dose, and accumulated dose were exported. Based on the DVH data, the max dose, the dose to 0.5–5cc in 0.5cc. increments were expressed as Dmax, and D0.5-D5cc.


$$\text{EQD}2=\text{D}x\;\frac{\frac{\alpha }{\beta }+{\text{d}}_{x}}{\frac{\alpha }{\beta }+{\text{D}}_{2}}$$

### Construction and validation of the NTCP model

Our NTCP model for temporal lobe was constructed based on multivariate logistic regression, formula of which is shown below as equation. x1, x2… xm are different input parameters; β0, β1… βm are the logistic regression coefficients of corresponding input parameters. Both dosimetric parameters and clinical factors were considered as potential input parameters in this model. Dosimetric parameters include D0.5cc-D5cc in 0.5cc increments and Dmax.


$$\text{NTCP}=\frac{1}{1+e^{-(a+\sum_{i=1}^{m}b_{i}x_{i})}}$$

All the patients were randomly divided into training set and validation set at a ratio of 2:1. Training set data were utilized for deriving model parameter, while validation set data were employed for accessing the model. Model construction involved two primary steps. Initially, three modes (linear, quadratic, exponential) were explored to assess the necessity of incorporating the time interval in the combination of doses from two courses. The verification process included the following steps: 1. Defining the range and stride of the parameter based on clinical data and time model formulation; 2. Applying parameter values to the time model to derive the combined dose; 3. Calculating dosimetric indices of the combined dose distribution; 4. Conducting univariate logistic analysis on the dosimetric index and obtaining Nagelkerke’s R squared value; 5. Repeating steps 2–4 for different parameter values and various time models.


$$\text{dose}\_\text{tol}={\left(1-{\text{a}}^{*}\text{gap}\right)}^{*}\text{dose}1+\text{dose}2$$

$$\text{dose}\_\text{tol}={\left(1-{\text{a}}^{*}\text{ ga}{\text{p}}^{*}\text{gap}\right)}^{*}\text{ dose}1+\text{dose}2$$

$$\text{dose}\_\text{tol}=\exp{\left(-{\text{a}}^{*}\text{ gap}\right)}^{*}\text{dose}1+\text{dose}2$$

Subsequently, multivariate logistic regression was conducted with different sets of factors. Considering diverse clinical scenarios, three protocols were presumed for broader application: 1st, the primary RT plan was unable to obtain, we only consider the 2nd RT plan dosage; 2nd, both the first and second RT plan were available, but we cannot calculate the accumulated dose; 3rd, both the RT plan were available and we use the accumulated EQD2 dose.

To validate the model, the area under the receiver operating characteristic curve (AUC) for the receiver operating characteristic curve (ROC) was calculated. Statistical analyses were performed using SPSS version 26.0 (IBA, SPSS Inc., Chicago, IL).

## Results

From Jan. 2010 to Dec. 2020, 78 patients were enrolled into our study. These patients were randomly divided into training data set and validation data set in a ratio of 2:1, yielding 52 patients in training set and 26 patients in validation set. There were no significant differences in clinical characteristics between these two sets. Characteristics of patients are listed in **Table 1**. The median follow-up time was 31 (range, 6–127) months. Among these patients, 26 (33.3%) developed TLI, among which 16 patients experiencing bilateral TLI and 10 patients with unilateral TL. The median latency for development of TLI (from beginning of re-irradiation to first MRI-detected TLI) was 11.5 (range, 3–29) months. The median interval between initial radiotherapy and re-irradiation was 26 (range, 12–108) months.

**Table 1:** Clinical characteristics of 78 patients with recurrent nasopharyngeal carcinoma.

Clinical characteristics		Training set No. (%)	Validation set No. (%)	P value
Gender				
Male		39 (75.0)	20 (76.9)	0.924
Female		13 (25.0)	6 (23.1)	
Median age (y) (range)		45 (29–66)	49 (30–62)	0.725
T stage of primary tumor				
1–2		29 (55.8)	12 (46.2)	0.728
3		14 (26.9)	7 (26.9)	
4		9 (17.3)	7 (26.9)	
N stage of primary tumor				
0–1		32 (61.5)	14 (53.8)	0.261
2–3		20 (38.5)	12 (46.1)	
RT dose of primary tumor				
66		23 (44.2)	10 (38.5)	0.493
70.4		29 (55.8)	16 (61.5)	
Interval between 1^st^ and 2^nd^ RT (range)		23.5 (12.1–108.3)	33.1 (13.8–84.4)	0.531
T stage of recurrent tumor				
0–2		31 (59.6)	12 (46.1)	0.742
3		16 (30.8)	10 (38.5)	
4		5 (9.6)	4 (15.4)	
N stage of recurrent tumor				
0		38 (73.1)	17 (65.4)	0.498
1		13 (25.0)	9 (34.6)	
2		1 (1.9)	0 (0)	
RT dose of recurrent tumor				
60		10 (19.2)	6 (23.1)	0.971
62		3 (5.8)	2 (7.7)	
64		3 (5.8)	1 (3.8)	
66		36 (69.2)	17 (65.4)	
Induction chemo	Yes	25 (48.1)	12 (46.2)	0.283
	No	27 (51.9)	14 (53.8)	
Concurrent chemo	Yes	13 (25.0)	5 (19.2)	0.373
	No	39 (75.0)	21 (80.8)	
Temporal lobe injury	Yes	16 (32.7)	10 (38.5)	0.774
	No	36 (67.3)	16 (61.5)	

**Figure 1:**
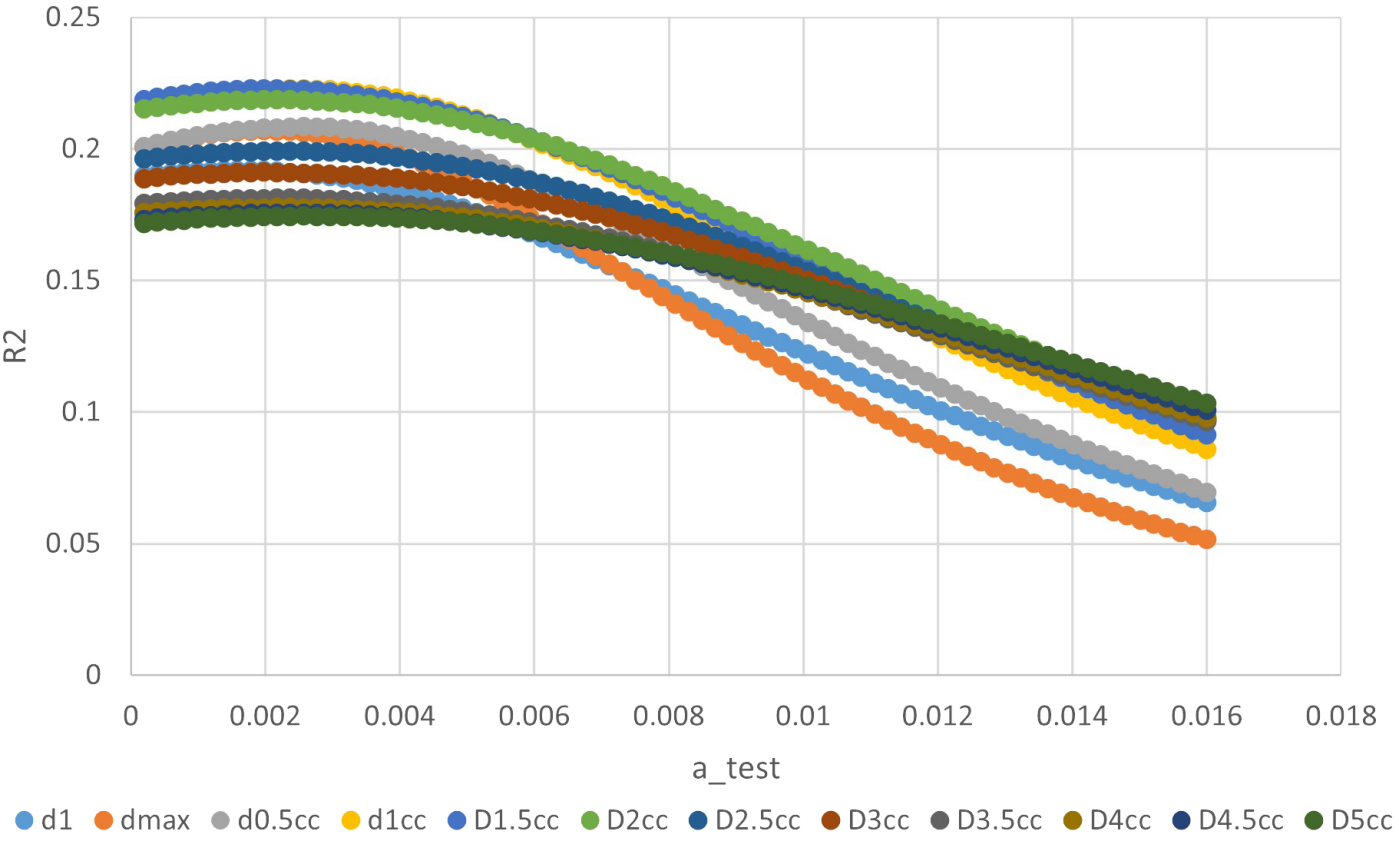
The relationship between ‘a’ value and R square of different indices in linear time model.

**Figure 2:**
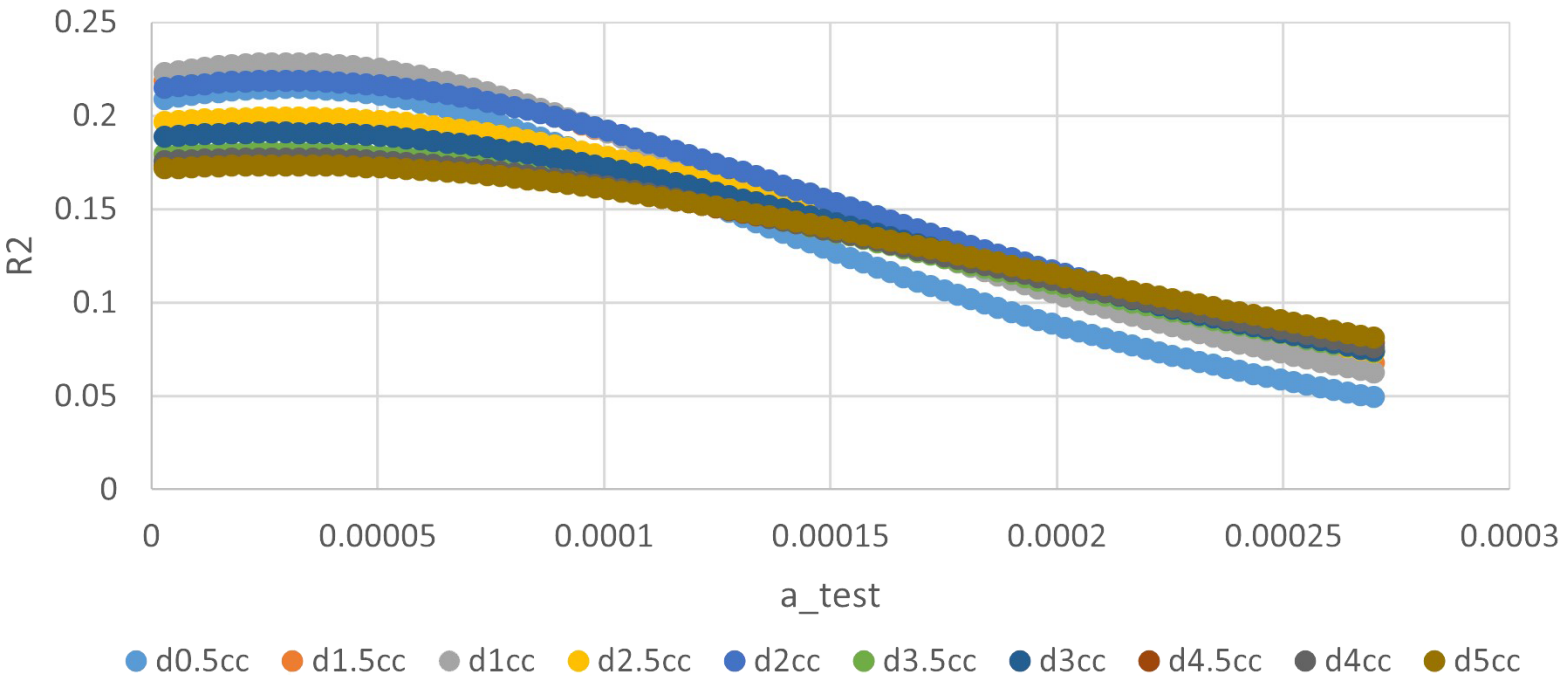
The relationship between ‘a’ value and R square of different indices in quadratic time model.

**Figure 3:**
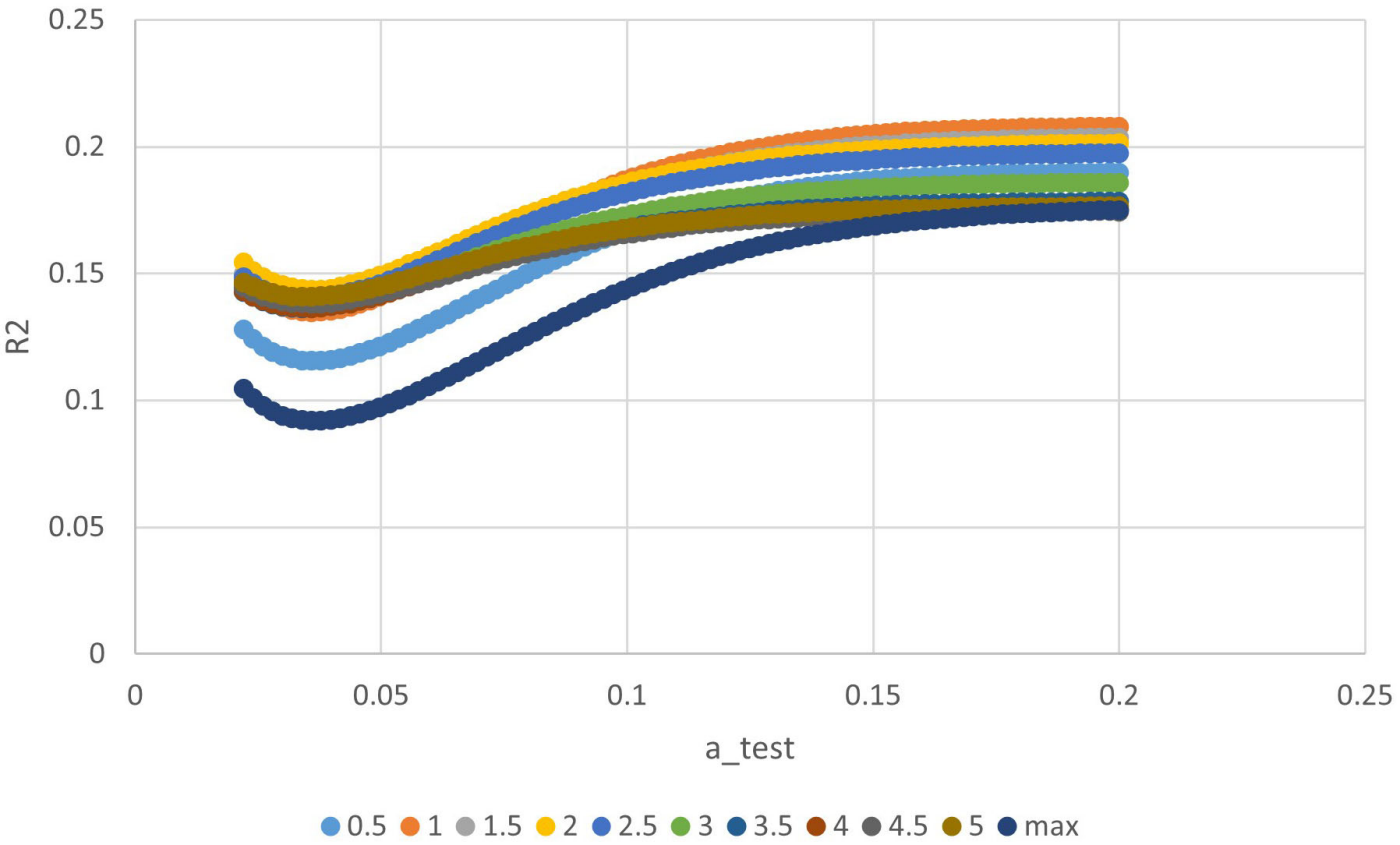
The relationship between ‘a’ value and R square of different indices in exponential time model.

The ‘a’ value corresponding to maximum R2 value for various indices in the linear time model was presented in **Figure 1**. It can be observed that the dose-volume parameter corresponding to the maximum R2 value is D1.5cc, as indicated in **Table 2**. Results of the ROC curve analysis, using the ‘a’ value corresponding to the maximum R2 value in the linear time model as the model parameter value, were displayed in **Table 3**. However, equation incorporating the time factor exhibited minimal deviation from the value obtained by straightforwardly summing temporal lobe doses voxel to voxel, as shown in **Figures 1**–**3**. Therefore, the necessity of incorporating interval time into the model was not clearly evident in our data.

**Table 2:** The ‘a’ value corresponding to maximum R2 value for different indices in linear time model.

	d1	dmax	d0.5cc	d1cc	D1.5cc	D2cc	D2.5cc	D3cc	D3.5cc	D4cc	D4.5cc	D5cc
R2_max	0.1920	0.2069	0.2084	0.2227	0.2228	0.2186	0.1993	0.1911	0.1813	0.1777	0.1755	0.1745
max_a	0.0018	0.0020	0.0026	0.0026	0.0022	0.0024	0.0022	0.0020	0.0024	0.0024	0.0022	0.0026

The volume‐dose parameter corresponding to the maximum R2 value is D1.5cc. The value of 0.2228 in the table represent the highest Nagelkerke’s R squared values obtained by testing various 'a' values and volume-dose parameters: applying different 'a' values to time models to calculate combined doses, computing dosimetric indices, and performing univariate logistic analysis.

**Table 3:** ROC curve analysis results with the ‘a’ value corresponding to maximum R2 value in linear time model as model parameter value.

Variable	Area under ROC curve	β	p	Lower- Upper limit	Cutoff point	Sensitivity	Specificity
Dmax	0.8241	0.04718	<0.000	0.7317–0.9166	125.6	74.19	92.47
D0.5	0.8144	0.04600	<0.0001	0.7243–0.9046	119.6	67.74	90.32
D1	0.8103	0.04559	<0.0001	0.7209–0.8996	116.0	67.74	89.25
D1.5	0.8068	0.04427	<0.0001	0.7200–0.8936	115.3	61.29	91.4
D2	0.8075	0.04392	<0.0001	0.7214–0.8936	113.1	58.06	92.47
D2.5	0.7971	0.04554	<0.0001	0.7078–0.8864	109.5	58.06	87.1
D3	0.7867	0.04646	<0.0001	0.6956–0.8777	94.48	80.65	63.44
D3.5	0.7829	0.04657	<0.0001	0.6916–0.8741	91.74	77.42	66.67
D4	0.7792	0.04674	<0.0001	0.6876–0.8708	89.60	77.42	67.74
D4.5	0.7784	0.04697	<0.0001	0.6863–0.8704	87.27	77.42	68.82
D5	0.7763	0.04743	<0.0001	0.6833–0.8692	84.06	77.42	69.89

**Figure 4:**
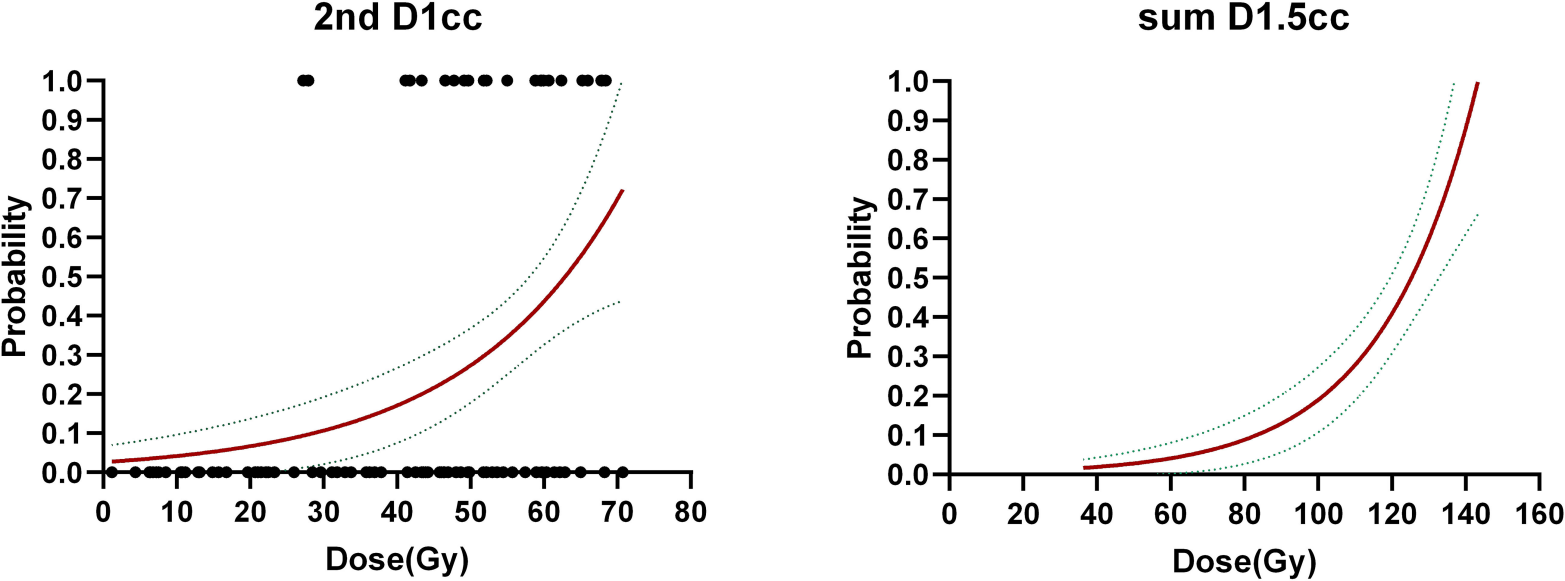
Dose-response curves for temporal lobe injury.

Multivariate NTCP model was derived by analyzing dosimetric variables, including relative dose delivered to specific volumes of temporal lobe (in 0.5 cc bins from Dmax to D5cc), and clinical factors, including primary and recurrent tumor stage, RT dose, tumor volume, time interval between two RT courses, chemotherapy, gender, and age. Possible clinical factors did not reach statistically significant differences in multivariate analysis, details were shown in **Table 4**. According to NTCP model, the TD5 and TD50 EQD2 re-RT dose of d1cc were 13.8Gy (0–20.35Gy), and 62.90Gy (42.49–80.93Gy), respectively. The TD5 and TD50 EQD2 dose of sum-dose d1.5cc were 65.26Gy(46.72–80.69Gy) and 125.25Gy(89.51–152.18Gy), respectively, see in **Figure 4**.

**Table 4:** Multivariate logistic regression analysis for temporal lobe injury.

	P value	Wald	95% CI	Regression function	Nagelkerke’s R squared
Only second course RT indices					
2^nd^ D1	<0.001	13.841	1.036–1.121	S=0.075×2^nd^D1–4.649	0.285
First and second radiation therapy indices, but without dose combination					
2^nd^D1	<0.001	12.190	1.032–1.117	S=0.071×2^nd^D1 + 0.084*1^st^Dmax -10.281	0.497
1^st^Dmax	0.030	4.688	1.008–1.173		
Indices of combined dose					
D1.5	<0.001	16.405	1.035–1.105	S=0.067×D1.5–8.216	0.330

**Figure 5:**
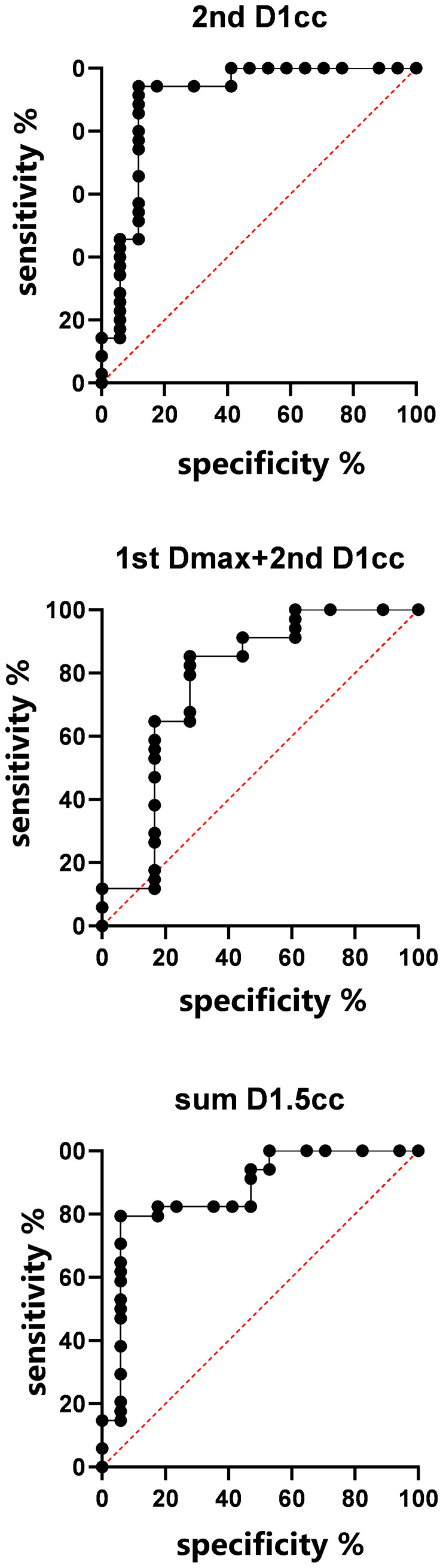
ROC curves for temporal lobe injury in validation set.

Model validation: If we consider the 2nd RT plan dose only, the AUC of the verification set was 0.9008, (0.7881–1), p< 0.001; If we consider the first and second RT plan dose individually, without considering their cumulative effect, the AUC was 0.7745(0.6199–0.9292), p=0.0012; For the accumulated EQD2 dose, the AUC was 0.8702 (0.7577–0.9828), p<0.001, as it’s shown in **Figure 5**.

## Discussion

With the widely use of three-dimensional conformal radiotherapy, dose-volume metrics are very important to understand and evaluate the tolerance of normal tissue to dose variation. Quantitative Analysis of Normal Tissue Effects in the Clinic (QUANTEC) (11) reported the relationship between the incidence of TLI and the volume dose of radiotherapy. The bioequivalent doses of TD5 and TD50 with TLI at a single dose< 2.5Gy were 120Gy (range: 100–140Gy) and 150Gy (range: 140–170Gy), respectively. However, it was generally believed that the tolerated dose of temporal lobe tissue was higher than the recommended reference dose of QUANTEC. There are very limited number of studies on the DVH probability of TLI after two-course radiotherapy for recurrent nasopharyngeal carcinoma. Lee et al. (12) analyzed 487 cases of nasopharyngeal carcinoma patients after re-radiotherapy, in which both primary and retreatment radiotherapy were two-dimensional radiotherapy. They found re-radiotherapy significantly increased the incidence of TLI. In the meta-analysis of re-radiotherapy of brain tissue (13), they found the two 2D radiotherapy treatments is safe when the sum of EQD2 is less than 96Gy, and the probability of TLI was 0–3% when the sum of EQD2 is less than 101Gy. Liu et al. (14) conducted dose-volumetric analysis on TLI of 227 patients with recurrent nasopharyngeal carcinoma. In these cases, the first course of radiotherapy was two-dimensional radiotherapy and the second course of radiotherapy was IMRT radiotherapy. However, in this study, the first temporal lobe dose was an estimated dose, and there is a question about the accuracy of directly summing two doses together.

Our study represents a comprehensive evaluation of the NTCP model for TLI in recurrent nasopharyngeal carcinoma. The rigorous approach includes the use of IMRT technology for both primary and recurrent treatment, dual RT plans?, and a minimum 6-month follow-up with available MRI images. Based on the NTCP model, the TD5 and TD50 of D1.5cc for the temporal lobe in our study, derived from the cumulative effect of two radiotherapy plans, was 65.3Gy and 125.3Gy, respectively. Notably, these values were more stringent compared to the EQD2-pMAX dose presented in Liu et al.’s study (14). This discrepancy may be attributed to the overlap of high-dose regions resulting from deformable image registration. TLI is often inevitable when dealing with recurrent tumors with re-irradiation, especially for recurrent tumors invading skull base or cavernous sinus. Consequently, minimizing temporal lobe exposure for the second course treatment planning is important. Consistent with prior research, our study revealed a shorter latency time for TLI post re-radiotherapy, with a median duration of 11.5 months.

The time interval between two radiotherapy treatments influences the incidence of temporal lobe injury, because with the extension of the interval time, the damage of normal tissue will recover gradually. The study by Liu et al. (14) found that the risk of TLI was significantly reduced in patients with nasopharyngeal carcinoma whose interval time was > 26 months. The study by Lee et al. (15) also found that the normal tissue tolerance of patients with an interval of more than two years had a trend of improvement compared with patients with recurrence within two years, but the difference was not statistically significant. Ang et al. (16) carried out two-course irradiation on the spinal cord of 56 macaque monkeys, and they believed that the spinal cord tolerance recovered significantly within one year after radiotherapy and gradually recovered further in the following years. In this study, we endeavored to formulate two radiotherapy dose superposition models by incorporating interval time variables in various approaches, such as linear, quadratic, and exponential time models. Our findings indicate that the equation incorporating the time factor exhibited minimal deviation from the value obtained by straightforwardly summing the temporal lobe doses voxel to voxel. Furthermore, no statistically significant difference was observed in the impact of interval time within the multifactor regression equation. Therefore, we believe that the two doses can be converted to EQD2 and then added on the corresponding voxels. The possible reason is, the minimum interval between the two radiotherapy treatments was 12 months, and the recovery of temporal lobe tissue was most obvious within one year.

MIM software was applied to map the first dose distribution to the second-course CT by registration of the two CTs. Python was applied to calculate and add EQD2 voxel by voxel. The rigid registration of these two CTs is challenging due to the relatively long interval between CT scans (median 28 months in this study). While there’s uncertainty in fusing MRI with CT, the rigid nature of the intracranial temporal lobe minimizes shape changes. The study’s method, utilizing MIM software for image fusion, is deemed effective in obtaining relatively accurate results under these circumstances (17, 18). To enhance clinical applicability, we addressed challenges such as the absence of the first-course treatment plan or the inability to accumulate initial and recurrent radiotherapy. In such cases, we employed corresponding models for calculations, extending the practicality of our study to accommodate diverse patient scenarios.

Beyond the considerations of volume-dose and interval time’s impact on TLI discussed above, correlation analysis was performed to investigate whether TLI was correlated with T stage, tumor volume, KPS score, gender, age, dose prescription, and administration of concurrent chemotherapy. No statistically significant difference was found in the correlation between these clinical factors and the occurrence of TLI. Su et al. (5) reported the incidence of TLI in nasopharyngeal carcinoma patients receiving chemo-radiotherapy is significantly higher than those of patients receiving radiotherapy alone. However, NPC patients received chemotherapy tend to be advanced stage, thus temporal lobe would be exposed to higher dose. In this study, patients with advanced T stage or larger tumor volume tended to have a higher proportion of TLI, but the differences were not statistically significant.

The study acknowledges several limitations, including a small sample size with both primary and secondary plans, inadequacy of follow-up duration (minimum six months in this study), a relatively low two-year overall survival rate (65%) which means that patients may passed away before developing TLI). Furthermore, developing a more comprehensive grading system for TLI grades is crucial, differentiating between mild (grades 1–2) and severe cases (grades 3–4). Achieving precision in these distinctions requires a larger sample size to ensure the accuracy of the results.

## Conclusion

In this study, an NTCP model of temporal lobe injury after re-IMRT radiotherapy for recurrent nasopharyngeal carcinoma was established. The most important factors affecting TLI was the sum-dose d1.5cc of TL. According to NTCP model, the TD5 and TD50 EQD2 dose of sum-dose d1.5cc were 65.26Gy (46.72–80.69Gy) and 125.25Gy (89.51–152.18Gy), respectively. When considering only the re-IMRT dose, the TD5 and TD50 EQD2 re-RT dose of d1cc for TLI were 13.8Gy (0–20.35Gy), and 62.90Gy (42.49–80.93Gy), respectively. Consequently, minimizing temporal lobe exposure during re-RT planning is crucial.

## Data availability statement

The original contributions presented in the study are included in the article/**Supplementary Material**. Further inquiries can be directed to the corresponding author.

## Ethics statement

Ethical approval was not required for the study involving humans in accordance with the local legislation and institutional requirements. Written informed consent to participate in this study was not required from the participants or the participants’ legal guardians/next of kin in accordance with the national legislation and the institutional requirements.

## Author contributions

XG: Conceptualization, Data curation, Funding acquisition, Investigation, Software, Writing – original draft, Writing – review & editing. JP: Data curation, Formal analysis, Investigation, Methodology, Software, Writing – review & editing. JS: Data curation, Formal analysis, Investigation, Methodology, Writing – review & editing. XX: Data curation, Funding acquisition, Writing – review & editing. CH: Conceptualization, Supervision, Validation, Writing – review & editing.
