# Supplementary material for: Normal tissue complication probability model of temporal lobe injury following re-irradiation of IMRT for local recurrent nasopharyngeal carcinoma
Source: Front Oncol. 2024 May 30;14:1394111. doi: 10.3389/fonc.2024.1394111 (PMC11169595; doi:10.3389/fonc.2024.1394111)
Supplement: Supplementary file 1 [file Presentation_1.pdf]

Supplementary Material

Part of the programming code involved in this study

- Read dicom files and clinical information to generate hdf5 and csv files

```
img_dir = DicomDirectory(dicom_path) #读取 dicom 文件
fp_data = read_fp_data_csv(fp_data_path) #读取其余临床信息

for    dose_series_dic,    dicom_rtst_series,    img_series_info in
img_dir.dose_rtst_and_its_img_series_iter():

with h5py.File(hdf5_path + os.sep + pt_id + ".h5", "w") as hf:
    hf.create_dataset("dose1", data=dose_img_coord1, compression='lzf')
    hf.create_dataset("dose2", data=dose_img_coord2, compression='lzf')
    hf.create_dataset("mask", data=slice_mask, compression='lzf')
    hf.create_dataset("roi_vol", data=roi_vol_ls, compression='lzf')

with open(hdf5_path + os.sep + "data_recoder.csv", 'a+') as csv_file:
    csv_file.write(pt_id+','+str(gap)+','+str(lobe_l_dmg)+','+str(lobe_r_dmg)+','+str(fr
ac_dose1)+','+str(frac_dose2)+','+'\n')
```

- The hdf5 and csv files are processed to obtain data for DVH calculations

```
for record_idx, record_item in enumerate(recoder_list):

    pt_id = record_item[0]
     pt_gap = record_item[1]
     lobe_l_dmg = record_item[2]
     lobe_r_dmg = record_item[3]
     frac_dose1 = record_item[4]
     frac_dose2 = record_item[5]

    dose1 = hdf5_file["dose1"][:]
    dose2 = hdf5_file["dose2"][:]
    mask = hdf5_file["mask"][:]
    roi_vol = hdf5_file["roi_vol"][:]

    lobe_l = mask[:, :, :, 0]
    lobe_r = mask[:, :, :, 1]
```

```
    dose1_lobe_l_ls = dose1[np.where(lobe_l == 1)]
    dose1_lobe_r_ls = dose1[np.where(lobe_r == 1)]

    dose1_lobe_l_np = np.array(dose1_lobe_l_ls)
    dose1_lobe_r_np = np.array(dose1_lobe_r_ls)

    dose1_eud2_lobe_l = dose1_lobe_l_np * (3 + dose1_lobe_l_np / int(frac_dose1)) / 5
    dose1_eud2_lobe_r = dose1_lobe_r_np * (3 + dose1_lobe_r_np / int(frac_dose1)) / 5

    dose2_lobe_l_ls = dose2[np.where(lobe_l == 1)]
    dose2_lobe_r_ls = dose2[np.where(lobe_r == 1)]

    dose2_lobe_l_np = np.array(dose2_lobe_l_ls)
    dose2_lobe_r_np = np.array(dose2_lobe_r_ls)

    dose2_eud2_lobe_l = dose2_lobe_l_np * (3 + dose2_lobe_l_np / int(frac_dose2)) / 5
    dose2_eud2_lobe_r = dose2_lobe_r_np * (3 + dose2_lobe_r_np / int(frac_dose2)) / 5

    lobe_l_vol = roi_vol[0]
    lobe_r_vol = roi_vol[1]

    yield dose1_eud2_lobe_l, dose2_eud2_lobe_l, lobe_l_dmg, lobe_l_vol, dose1_eud2_lobe_r, dose2_eud2_lobe_r, lobe_r_dmg, lobe_r_vol, pt_gap
    # yield dose1_lobe_l_np, dose2_lobe_l_np, lobe_l_dmg, lobe_l_vol, dose1_lobe_r_np, dose2_lobe_r_np, lobe_r_dmg, lobe_r_vol, pt_gap
```

- Computed DVH

```
def dvh_cal(cfg,a,dvh_para,dose_method):
    dvh_stat_ls = []
    dmg_ls = []
    for l1, l2, l_dmg, l_vol, r1, r2, r_dmg, r_vol, gap in SegData(cfg):
        if dose_method==0:
            l_tol = l1 + l2
        if dose_method==1:
            l_tol = l1
        if dose_method==2:
            l_tol = l2
        if len(l_tol) != 0:
            l_dvh = DVH.from_data(l_vol, l_tol, binsize=0.01)
```

```
                if dvh_para == 'Dmax':
                    l_dvh_stat = l_dvh.max
                else:
                    l_dvh_stat = l_dvh.statistic(dvh_para)
                dvh_stat_ls.append(l_dvh_stat)
                dmg_ls.append(int(l_dmg))
            if dose_method == 0:
                r_tol = r1 + r2
            if dose_method == 1:
                r_tol = r1
            if dose_method == 2:
                r_tol = r2
            if len(r_tol) != 0:
                r_dvh = DVH.from_data(r_vol, r_tol, binsize=0.01)
                if dvh_para == 'Dmax':
                    r_dvh_stat = r_dvh.max
                else:
                    r_dvh_stat = r_dvh.statistic(dvh_para)
                dvh_stat_ls.append(r_dvh_stat)
                dmg_ls.append(int(r_dmg))
    return dvh_stat_ls, dmg_ls
```

- The superposition mode of DVH at different time was calculated, and the left and right temporal lobes were processed separately

```
dose_tol = (1-a*float(gap)) * dose1+dose2
dose_tol = (1-a* float(gap) * float(gap)) * dose1+dose2
dose_tol = np.exp(-a*float(gap)) * dose1 + dose2
```

- Pseudo-R square curves was got from different a values and different DVH parameters

```
def logi_cal(dvh_stat_ls,dmg_ls):
    dvh_stat_np = np.array(dvh_stat_ls)
    dmg_np = np.array(dmg_ls)
    dvh_stat_np = sm.add_constant(dvh_stat_np)
    lr = sm.Logit(dmg_np, dvh_stat_np)
    result = lr.fit()
    return result.prsquared

def dose_cal(cfg):
    from dose_cal import dvh_cal, logi_cal
```

```
        from utilities import change_dot
        a_lower = cfg.a_lower
        a_upper = cfg.a_upper
        length = cfg.length
        output_folder = to_abs_path(cfg.output_path)
        for i in
['D0.5cc','D1cc','D1.5cc','D2cc','D2.5cc','D3cc','D3.5cc','D4cc','D4.5cc''D5cc','Dmax']:
                output_file = output_folder+ i +'.csv'
                with open(output_file, 'a+') as csv_file:
                        for a in [a_lower + x * (a_upper - a_lower) / length for x in
range(length)]:
                                dvh_stat_ls, dmg_ls = dvh_cal(cfg, a, i)
                                p_r2 = logi_cal(dvh_stat_ls, dmg_ls)
                                csv_file.write(str(a) + ',' + str(p_r2) + ',' + '\n')
```

- DVH results and temporal lobe injury output

```
def dose_test(cfg):
        from dose_cal import dvh_cal
        from utilities import change_dot
        import csv
        a = 0
        output_folder = to_abs_path(cfg.output_path)
        dvh_para_ls =
['D0.5cc','D1cc','D1.5cc','D2cc','D2.5cc','D3cc','D3.5cc','D4cc','D4.5cc''D5cc','Dmax']
        dose_type_ls = [0,1,2]
        dvh_dose_ls = [[dvh_type, dose_type] for dvh_type in dvh_para_ls for
dose_type in dose_type_ls]
        for dvh_para, dose_method in dvh_dose_ls:
                output_file = output_folder + 'eqd2' + '_'+str(dose_method)
+'_'+str(dvh_para) + '.csv'
                with open(output_file, 'a+') as csv_file:
                        csv_file.write('eqd2'+','+'damage'+','+'\n')
                        dvh_stat_ls, dmg_ls = dvh_cal(cfg, a, dvh_para, dose_method)
                        for dvh_out in range(len(dvh_stat_ls)):
                            csv_file.write(str(dvh_stat_ls[dvh_out]) + ',' + str(dmg_ls[dvh_out])
+ ',' + '\n')
```

- model test

```
def model_test(cfg):
        from dose_cal import dvh_cal
        output_folder = to_abs_path(cfg.output_path)
        output_file = output_folder + 'test_photon_dose2_d1cc'+ '.csv'
```

```
        dvh_val1_ls, dmg_ls = dvh_cal(cfg, 0, 'D1cc', 2)
        # dvh_val2_ls, dmg_ls = dvh_cal(cfg, 0, 'Dmax', 1)
        # dvh_val_ls, dmg_ls = dvh_cal(cfg, 0, 'D1.5cc', 0)
        model_out_ls=[]
        for dvh_val_index in range(len(dmg_ls)):
                s=0.075*dvh_val1_ls[dvh_val_index]-4.649
                model_out=model_cal(s)
                if model_out>1 or model_out<0:
                        logger.info('something wrong!')
                        break
                model_out_ls.append(model_out)
        with open(output_file, 'a+') as csv_file:
                csv_file.write('dose2_d1cc' + ',' + 'damage' + ',' + '\n')
                for out_index in range(len(dmg_ls)):
                        csv_file.write(str(model_out_ls[out_index]) + ',' +
str(dmg_ls[out_index]) + ',' + '\n')

def model_cal(s):
        import math
        y=math.exp(-1*s)
        y=1/(1+y)
        return y
```
